# Extracapsular and joint-dominant hand and wrist MRI patterns in psoriatic and rheumatoid arthritis: an exploratory case-control study

**DOI:** 10.3389/fimmu.2026.1945180

**Published:** 2026-10-20

**Authors:** Juan Huang, Shaohui Song, Quanhong Zhang, Qiuyu Fan, Yue Li, Yiqian Peng, Yue Zhu, Gang Hu, Lang Yu, Jingru Chen, Liuqing Chen, Ping Xia, Jinbo Chen

**Affiliations:** 1 Hubei University of Chinese Medicine, Wuhan, China; 2 Department of Dermatology, Traditional Chinese and Western Medicine Hospital, Hubei University of Chinese Medicine, Wuhan, China; 3 Department of Dermatology, Wuhan No. 1 Hospital, Wuhan, China; 4 Department of Radiology, Wuhan No. 1 Hospital, Wuhan, China; 5 Tongji Medical College, Huazhong University of Science and Technology, Wuhan, China; 6 Department of Rheumatology and Immunology, Wuhan No. 1 Hospital, Wuhan, China; 7 School of Medicine, Jianghan University, Wuhan, China; 8 Hubei Province and Key Laboratory of Skin Infection and Immunity, Hubei Provincial Clinical Research Center for Infectious Skin Diseases, Wuhan, China; 9 Dermatology Hospital, Southern Medical University, Guangdong, China

**Keywords:** enthesitis, imaging phenotype, inflammatory arthritis, magnetic resonance imaging, psoriatic arthritis, rheumatoid arthritis, synovio-entheseal complex

## Abstract

**Background:**

Psoriatic arthritis (PsA) and rheumatoid arthritis (RA) may present with overlapping hand and wrist symptoms, while their anatomical patterns of inflammation differ.

**Objective:**

To compare prespecified hand and wrist magnetic resonance imaging (MRI) features between PsA and RA and to assess selected exploratory associations, discriminatory performance, anatomical distributions and reader reliability.

**Methods:**

This retrospective case-control study included 41 patients with PsA and 41 with RA who underwent clinically acquired 3.0-T hand or wrist MRI. Research MRI features were assigned retrospectively by readers blinded to the clinical diagnosis. Between-group comparisons used available-case denominators. Each adjusted model included one MRI feature together with age, sex and symptom duration; Firth bias-reduced logistic regression was used for the sparse vascular-like signal feature.

**Results:**

Compared with RA, PsA had higher median scores for extracapsular soft tissue inflammation (7 vs. 2), periarthritis (8 vs. 4) and bead-like peritendinous effusion (1 vs. 0; all P <0.01). Study-defined prominent reticular vascular-like signal occurred in 35/41 patients with PsA and 2/41 with RA and showed a strong but imprecise adjusted association with PsA (odds ratio 85.02, 95% CI 15.07–479.83). RA had a higher median joint effusion score (5 vs. 2) and more frequent wrist synovitis (33/41 vs. 7/37) and wrist bone erosion (30/41 vs. 8/39; all P <0.01). Inter-reader coefficients ranged from 0.574 to 0.914 and intra-reader coefficients from 0.846 to 0.968.

**Limitations:**

The historical screening denominator and formal composite disease activity scores could not be reconstructed; one clinically diagnosed RA case did not meet the retrospective classification threshold; treatment exposure differed between groups; distal interphalangeal and ulnocarpal coverage was incomplete; and external validation was not performed.

**Conclusion:**

In this exploratory cohort, extracapsular and enthesis-associated MRI findings favored PsA, whereas joint effusion and wrist synovitis or erosion favored RA. The study-defined vascular-like and bead-like features require independent validation before clinical use.

## Highlights

- Compared with rheumatoid arthritis, psoriatic arthritis showed more extracapsular and enthesis-associated hand or wrist MRI findings, whereas rheumatoid arthritis showed more wrist synovitis, wrist bone erosion and joint effusion.
- A study-defined reticular vascular-like signal separated the groups in this cohort but had only moderate inter-reader agreement and requires external validation.

## Introduction

Psoriatic arthritis (PsA) and rheumatoid arthritis (RA) commonly involve the hand and wrist and may present with overlapping pain, swelling and functional limitation (1, 2). Patients with psoriasis may first present in dermatology before a musculoskeletal diagnosis is established, supporting systematic screening in dermatology settings (3–5). Early differentiation is clinically important because delayed recognition of PsA is associated with structural and functional harm (6, 7).

Magnetic resonance imaging (MRI) can demonstrate synovitis, bone marrow edema (BME)/osteitis, tenosynovitis and periarticular inflammation and can detect structural damage earlier than conventional radiography in selected settings (8, 9). Outcome Measures in Rheumatology (OMERACT)/Rheumatoid Arthritis Magnetic Resonance Imaging Score (RAMRIS) and published tenosynovitis frameworks provide standardized terminology and scoring principles for several of these abnormalities (10–12). Comparative hand and wrist MRI literature—including a multimodality review and direct comparative cohorts—has generally associated extracapsular, periarticular and enthesis-related abnormalities with PsA, whereas wrist and joint-centered synovitis and erosion are more frequent in RA (1, 13–15). The synovio-entheseal complex provides an anatomical framework for understanding enthesis-associated, tendon and capsular inflammation, but the studies establishing that concept are not direct comparative MRI evidence (16, 17).

Histopathological studies describe differences in synovial vascularity, angiogenesis and vascular morphology between PsA and RA (18–20). These findings provide biological context but do not validate a disease-specific MRI vascular biomarker. Any vascular-like MRI pattern therefore requires an explicit operational definition, reproducibility assessment and external validation.

The primary objective of this study was to compare prespecified individual hand and wrist MRI features between clinically diagnosed PsA and RA. Secondary exploratory objectives were to evaluate feature-specific adjusted associations, individual-feature discrimination, anatomical distribution and reader reliability. We specifically examined two study-defined features—bead-like peritendinous effusion and prominent reticular vascular-like signal—within a broader descriptive framework of extracapsular or enthesis-associated versus joint-dominant or intracapsular findings.

## Methods

### Study design and ethical compliance

This single-center retrospective case-control study included patients with clinically diagnosed PsA or RA who underwent hand or wrist MRI for musculoskeletal symptoms at Wuhan No. 1 Hospital between 2021 and 2025. MRI examinations were acquired during routine care; blinded retrospective image assessment and statistical analysis were conducted after ethics approval. The report follows the Strengthening the Reporting of Observational Studies in Epidemiology (STROBE) reporting framework. The Medical Ethics Committee of Wuhan No. 1 Hospital (Wuhan Hospital of Integrated Traditional Chinese and Western Medicine) approved the study (approval number [2025] 151). Patients and the public were not involved in the study design, conduct, reporting or dissemination plans.

### Source population, eligibility, and diagnostic classification

Electronic medical records and the picture archiving and communication system were retrospectively searched for patients with a rheumatologist-documented diagnosis of PsA or RA who had undergone hand or wrist MRI for musculoskeletal symptoms during the study period. Exclusion criteria were incomplete or technically inadequate MRI, inability to confirm the clinical diagnosis from the record, or an alternative inflammatory, infectious, traumatic or crystal-related arthropathy that better explained the hand or wrist symptoms. The retained source files did not include a historical screening log; consequently, the number initially screened, reason-specific exclusion counts and whether all eligible examinations were enrolled consecutively could not be reconstructed. The final analyzable case-control sample comprised 41 patients in each group.

The original rheumatologist-recorded clinical diagnosis defined study-group membership. Classification criteria were applied retrospectively as a descriptive verification step: PsA components were assessed according to the Classification Criteria for Psoriatic Arthritis (CASPAR) (21), and available RA components were assessed according to the 2010 American College of Rheumatology/European League Against Rheumatism criteria (22). For RA threshold verification, recorded joint-involvement and duration components were combined with available serology and acute-phase-reactant data. When a qualitative rheumatoid factor (RF) or anti-cyclic citrullinated peptide antibody (anti-CCP) result was positive but the corresponding level relative to the upper limit of normal was unavailable, the lowest applicable positive serology score (2 points) was used. When an unavailable acute-phase-reactant component could not change whether the minimum possible total met the 6-point threshold, threshold status was assigned from that minimum but no exact total was assigned. A patient whose retrospectively reconstructed score was below 6 remained in the RA group when the contemporaneous rheumatology record supported the diagnosis. Because this was a retrospective study, the medical records did not permit consistent reconstruction of whether routine clinical MRI contributed to each patient’s original clinical diagnosis. The research MRI scores and study-defined MRI features were assigned retrospectively by blinded readers and were not used to define the study groups.

### Clinical, laboratory, and treatment variables

Age, sex, duration of musculoskeletal symptoms at MRI, RF, anti-CCP, erythrocyte sedimentation rate (ESR), C-reactive protein (CRP) and systemic treatment at MRI were abstracted from the records. Laboratory variables were summarized among tested patients, with the denominator shown for every comparison. The records did not consistently contain the tender and swollen joint counts, patient global assessment or other components required to reconstruct the Disease Activity Index for Psoriatic Arthritis (DAPSA) or Disease Activity Score in 28 joints (DAS28). ESR and CRP were therefore treated as limited inflammatory markers and not as substitutes for composite disease activity.

Systemic treatment at MRI was grouped as glucocorticoid, conventional synthetic disease-modifying antirheumatic drug (csDMARD), Janus kinase (JAK) inhibitor or biologic therapy. A patient could contribute to more than one category. Dose, treatment duration, adherence and prior treatment history were not sufficiently complete for comparative modeling.

### MRI acquisition, coverage, and blinded review

Hand or wrist MRI was performed on a 3.0-T scanner (MAGNETOM Vida, Siemens Healthineers) with a dedicated hand coil. The clinical protocol included coronal T1-weighted and T2-weighted spin-echo sequences, axial and sagittal fat-suppressed T2-weighted sequences and coronal three-dimensional proton-density sampling perfection with application-optimized contrasts using different flip angle evolutions (SPACE). No contrast-dependent research feature was used.

The principal field of view covered the radiocarpal, midcarpal, carpometacarpal, metacarpophalangeal (MCP), proximal interphalangeal and thumb interphalangeal joints. The distal radioulnar joint was recorded during wrist re-review. Evaluated tendon regions included the flexor tendon sheaths and wrist extensor compartments when covered. Distal interphalangeal joints were not consistently included across examinations and were not used for stable site-level comparisons. The ulnocarpal compartment was not assigned a separate score.

Two readers with musculoskeletal imaging experience independently assessed the MRIs while blinded to clinical diagnosis and other clinical information; disagreements in the main dataset were resolved by consensus. After the 82-case study dataset had been established, a 60-examination subset (30 PsA and 30 RA) underwent separate repeatability assessment. Reader 1 and reader 2 scores were used for inter-reader analysis, and the first and repeat assessments by reader 1 were used for intra-reader analysis. The repeatability exercise did not alter the main consensus dataset.

### MRI features and operational definitions

Terminology for BME/osteitis and bone erosion was informed by OMERACT/RAMRIS definitions, and tenosynovitis terminology was informed by the published MRI tenosynovitis framework (10–12). Because the clinical protocol did not include a standardized post-gadolinium sequence, the original contrast-dependent RAMRIS synovitis and tenosynovitis scoring definitions were not applied. Synovitis was therefore defined for this study as inflammatory thickening or abnormal signal of synovial tissue on the available non-contrast sequences and was recorded separately from joint effusion. BME/osteitis was inflammatory water-sensitive signal within bone marrow. Bone erosion required cortical interruption with adjacent osseous abnormality. Joint effusion was intra-articular fluid signal distinct from synovial thickening. Tenosynovitis was fluid and/or synovial-type inflammatory change within a tendon sheath. Study-specific semiquantitative features were graded from 0 to 3 in each evaluable predefined region, with 0 denoting absence and scores of 1 to 3 denoting increasing extent or severity, and were summed at patient level. The eligible regions were those listed in the MRI coverage subsection. Regions not captured within the field of view were treated as unavailable rather than normal. These retrospective study-specific sums were not interpreted as formally validated RAMRIS total scores.

Extracapsular soft tissue inflammation was diffuse or patchy inflammatory edema outside the joint capsule on fluid-sensitive sequences. Enthesitis was focal inflammatory change at a tendon, ligament or pulley attachment, with or without adjacent peri-entheseal edema. In the supplemental site review, enthesitis was recorded as present or absent in MCP and interphalangeal regions; these site indicators described distribution and did not replace the original summed enthesitis score. Periarthritis was inflammation involving the periarticular capsule and immediately adjacent extracapsular soft tissues and was distinguished from isolated tenosynovitis.

Bead-like peritendinous effusion was a study-defined finding consisting of discontinuous, nodular or segmental fluid collections along the tendon sheath or pulley system; continuous linear or fusiform sheath fluid was not classified as bead-like. The feature was scored by region and summed in the main analysis and was assessed as present or absent in the reliability exercise. Prominent reticular vascular-like signal was a study-defined binary feature consisting of irregular reticular or clustered vascular-like signal in synovial or periarticular tissue on non-contrast sequences, reproducible on adjacent slices and not following a normal straight vessel course. It was not interpreted as histological vascular hyperplasia or as a validated biomarker.

Wrist inflammation and structural damage were analyzed separately. Wrist synovitis was recorded in the radiocarpal, midcarpal and distal radioulnar joints on a 0–3 scale and classified as present if any evaluable region scored above 0; a patient was classified as absent only when all three regions were evaluable and scored 0. Wrist bone erosion was recorded as present or absent in the same three regions and summarized using the corresponding available-case rule. The earlier composite concept of “wrist involvement” was not used because it combined active inflammation with structural damage. Patient-level dactylitis and combined tenosynovitis-plus-dactylitis outcomes were not retained in the revised analyses because their values could not be reconstructed consistently from the retrospective patient-level record.

### Study objectives and outcomes

The primary outcome was the between-group distribution or frequency of each prespecified individual MRI feature. Secondary exploratory outcomes were: (1) the association of each MRI feature with PsA after adjustment for age, sex and symptom duration; (2) the area under the receiver operating characteristic curve (AUC) for selected individual features; (3) MCP and interphalangeal enthesitis distribution; and (4) inter- and intra-reader reliability. No externally validated diagnostic rule was prespecified, and no combined diagnostic model was retained in the revised analysis.

### Statistical analysis

Age is reported as mean ± standard deviation and was compared with an independent-samples t-test. Symptom duration and bounded MRI scores are reported as median (interquartile range [IQR]) and were compared with the Mann–Whitney U test. Categorical variables are reported as n/N (%) when data were incomplete and otherwise as n (%); chi-square or two-sided Fisher’s exact tests were used as appropriate. Analyses used available cases without imputation. P-values are two-sided, with P <0.05 considered statistically significant; because the analyses were exploratory, no multiplicity adjustment was made.

For each MRI feature, a separate logistic regression model used PsA (rather than RA) as the outcome and included the feature, age, sex and symptom duration. Standard maximum-likelihood logistic regression was used when it converged; Firth bias-reduced logistic regression was used for prominent reticular vascular-like signal because of quasi-separation. Odds ratios (ORs) for scores refer to a one-point increase. Model sample size and method are reported in **Table 1**. Because one clinically diagnosed RA patient did not meet the retrospectively assessed 6-point classification threshold, a *post-hoc* sensitivity analysis repeated the between-group comparisons and feature-specific models after excluding that patient.

**Table 1:** Feature-specific logistic regression models for association with psoriatic arthritis.

MRI feature	N	Method	Adjusted OR (95% CI)	P
Periarthritis score	82	Standard	1.22 (1.06–1.40)	0.005
Extracapsular soft tissue inflammation score	82	Standard	1.13 (1.01–1.27)	0.028
Joint effusion score	82	Standard	0.81 (0.69–0.95)	0.008
Enthesitis score	82	Standard	1.45 (0.93–2.24)	0.099
Bead-like peri-tendinous effusion score	82	Standard	2.05 (1.22–3.47)	0.007
BME/osteitis score	82	Standard	0.97 (0.82–1.15)	0.749
Tenosynovitis score	82	Standard	1.05 (0.96–1.15)	0.286
Prominent reticular vascular-like signal	82	Firth	85.02 (15.07–479.83)	<0.001
Wrist synovitis	78	Standard	0.08 (0.02–0.26)	<0.001
Wrist bone erosion	80	Standard	0.11 (0.04–0.36)	<0.001

BME, bone marrow edema; CI, confidence interval; MRI, magnetic resonance imaging; OR, odds ratio. Each row is a separate model with PsA (vs. RA) as the outcome and includes the listed MRI feature, age, sex and symptom duration. ORs for scores are per one-point increase. Standard indicates maximum-likelihood logistic regression; Firth indicates bias-reduced logistic regression. Analyses used available cases without imputation.

AUCs were estimated for selected individual features in the direction favoring PsA or RA, as indicated, and 95% CIs were obtained from 10,000 group-stratified bootstrap samples. Reliability of continuous scores was assessed with a two-way random-effects, absolute-agreement, single-measure intraclass correlation coefficient [ICC(2,1)]; binary features were assessed with unweighted Cohen’s kappa. Calculations for the revision were independently reproduced from the patient-level source tables using Python 3.12.14.

## Results

### Patient selection, classification, and clinical characteristics

The final analysis included 82 patients: 41 with PsA and 41 with RA. The unavailable historical screening log precluded reporting an initial screening denominator or reason-specific exclusion counts. All 41 PsA patients retrospectively had a CASPAR score of at least 3 (range 3–5). In the clinically diagnosed RA group, joint-involvement and duration components were available for 41/41 patients, at least one serological result for 41/41 and at least one acute-phase-reactant result for 40/41. Based on exact component scores where recorded and conservative minimum scores where an exact serology or acute-phase component was unavailable, 40/41 RA patients met the 2010 American College of Rheumatology (ACR)/European League Against Rheumatism (EULAR) threshold of at least 6. One patient had a retrospectively reconstructed score of 5 and was retained on the basis of the rheumatologist-documented clinical diagnosis. For patients in whom the available categories established a score range rather than an exact total, threshold status was determined from the minimum possible score. A *post-hoc* sensitivity analysis excluding the patient with a score of 5 did not materially change the direction or statistical significance of the primary between-group comparisons or feature-specific adjusted associations.

Mean age did not differ significantly between groups (RA 55.27 years ± 14.27 years vs. PsA 49.30 ± 14.17 years; P = 0.061). Female sex was more frequent in RA (31/41 [75.6%]) than PsA (14/41 [34.1%]; P <0.001). Median symptom duration at MRI was 2.6 months (IQR 2.2–3.0) in RA and 1.5 months (IQR 1.1–8.0) in PsA (P = 0.001). RF and anti-CCP positivity were more frequent in RA, whereas the proportions with elevated ESR and CRP did not differ significantly among tested patients (**Table 2**).

**Table 2:** Clinical, laboratory, treatment and MRI characteristics of patients with psoriatic arthritis and rheumatoid arthritis.

Variable	RA (n = 41)	PsA (n = 41)	P
Clinical and demographic characteristics			
Age, years, mean ± SD	55.27 ± 14.27	49.30 ± 14.17	0.061
Female sex, n (%)	31 (75.6)	14 (34.1)	<0.001
Symptom duration at MRI, months, median (IQR)	2.6 (2.2–3.0)	1.5 (1.1–8.0)	0.001
Laboratory results among tested patients			
RF positive, n/N (%)	33/37 (89.2)	0/30 (0)	<0.001
anti-CCP positive, n/N (%)	24/29 (82.8)	1/19 (5.3)	<0.001
ESR elevated, n/N (%)	21/30 (70.0)	12/24 (50.0)	0.167
CRP elevated, n/N (%)	20/29 (69.0)	14/26 (53.8)	0.279
Systemic treatment at MRI			
Glucocorticoid, n (%)	32 (78.0)	4 (9.8)	<0.001
csDMARD, n (%)	25 (61.0)	12 (29.3)	0.007
JAK inhibitor, n (%)	8 (19.5)	13 (31.7)	0.312
Biologic therapy, n (%)	6 (14.6)	12 (29.3)	0.181
MRI features			
Extracapsular soft tissue inflammation score, median (IQR)	2 (1–4)	7 (4–10)	<0.001
Enthesitis score, median (IQR)	0 (0–0)	0 (0–1)	0.003
Bead-like peri-tendinous effusion score, median (IQR)	0 (0–1)	1 (1–2)	0.002
Periarthritis score, median (IQR)	4 (2–6)	8 (6–11)	<0.001
Joint effusion score, median (IQR)	5 (3–8)	2 (1–6)	0.003
Tenosynovitis score, median (IQR)	1 (1–4)	3 (1–8)	0.123
BME/osteitis score, median (IQR)	0 (0–2)	1 (0–2)	0.604
Wrist synovitis, n/N (%)	33/41 (80.5)	7/37 (18.9)	<0.001
Wrist bone erosion, n/N (%)	30/41 (73.2)	8/39 (20.5)	<0.001
Prominent reticular vascular-like signal, n/N (%)	2/41 (4.9)	35/41 (85.4)	<0.001
Site-level enthesitis			
MCP-region enthesitis, n/N (%)	13/41 (31.7)	23/40 (57.5)	0.026
Interphalangeal-region enthesitis, n/N (%)	0/41 (0)	21/40 (52.5)	<0.001

anti-CCP, anti-cyclic citrullinated peptide antibody; BME, bone marrow edema; CRP, C-reactive protein; csDMARD, conventional synthetic disease-modifying antirheumatic drug; ESR, erythrocyte sedimentation rate; IQR, interquartile range; JAK, Janus kinase; MCP, metacarpophalangeal; MRI, magnetic resonance imaging; PsA, psoriatic arthritis; RA, rheumatoid arthritis; RF, rheumatoid factor; SD, standard deviation. Percentages use the displayed available denominator. Age was compared with an independent-samples t test; symptom duration and MRI scores with Mann–Whitney U tests; and categorical variables with chi-square or two-sided Fisher’s exact tests as appropriate. Treatment categories were not mutually exclusive.

Treatment at MRI differed between groups. Glucocorticoid exposure was recorded in 32/41 (78.0%) RA and 4/41 (9.8%) PsA patients (P <0.001), and csDMARD exposure in 25/41 (61.0%) and 12/41 (29.3%), respectively (P = 0.007). JAK inhibitor and biologic exposure did not differ significantly (**Table 2**). These treatment comparisons are descriptive because indication, dose and duration were not available for adequate confounding control.

### Between-group MRI comparisons

Compared with RA, PsA had higher scores for extracapsular soft tissue inflammation (median 7 [IQR 4–10] vs. 2 [1–4]; P <0.001), enthesitis (0 [0–1] vs. 0 [0–0]; P = 0.003), bead-like peritendinous effusion (1 [1–2] vs. 0 [0–1]; P = 0.002) and periarthritis (8 [6–11] vs. 4 [2–6]; P <0.001). The identical median enthesitis value reflects the large number of zero scores; the rank distributions differed. Prominent reticular vascular-like signal was present in 35/41 (85.4%) PsA and 2/41 (4.9%) RA examinations (P <0.001).

RA had a higher joint effusion score than PsA (5 [3–8] vs. 2 [1–6]; P = 0.003). Wrist synovitis was present in 33/41 (80.5%) evaluable RA and 7/37 (18.9%) evaluable PsA examinations, and wrist bone erosion in 30/41 (73.2%) and 8/39 (20.5%), respectively (both P <0.001). Tenosynovitis and BME/osteitis scores did not differ significantly. In the site-level review, MCP-region enthesitis was present in 13/41 RA and 23/40 PsA examinations (P = 0.026), while interphalangeal-region enthesitis was present in 0/41 and 21/40, respectively (P <0.001) (**Table 2**). Representative MRI appearances are shown in **Figure 1**.

**Figure 1:**
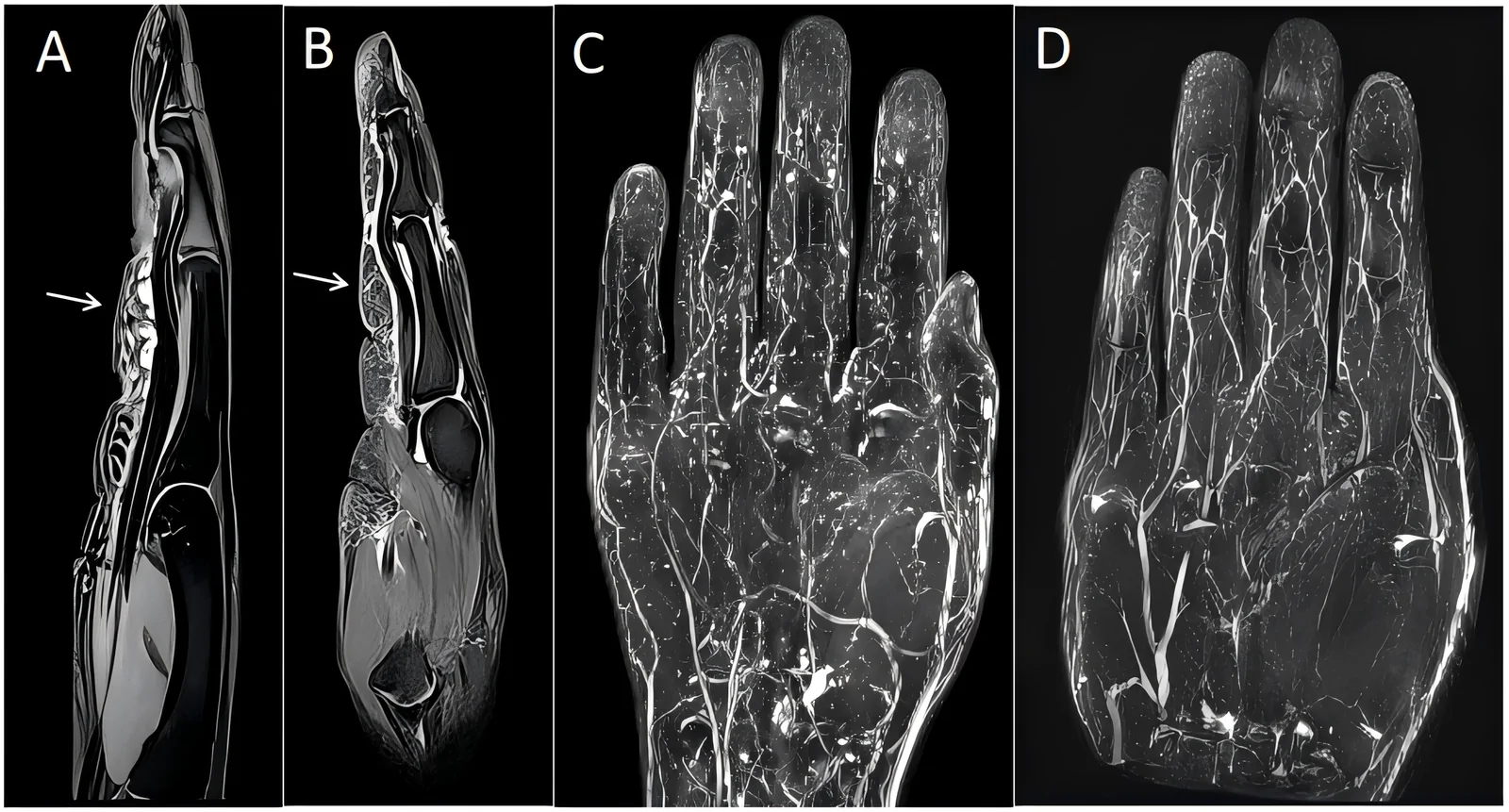
Representative hand and wrist MRI features. **(A)** Psoriatic arthritis showing study-defined bead-like peritendinous effusion, with discontinuous nodular or segmental fluid collections along the tendon sheath or pulley system. **(B)** Rheumatoid arthritis comparator showing more continuous linear or fusiform tendon-sheath fluid. **(C)** Psoriatic arthritis showing study-defined prominent reticular vascular-like signal, with irregular reticular or clustered signal in synovial or periarticular tissue. **(D)** Rheumatoid arthritis comparator with less clustered vascular-like signal.

### Feature-specific adjusted associations

In separate models adjusted for age, sex and symptom duration, higher periarthritis, extracapsular soft tissue inflammation and bead-like peritendinous effusion scores were associated with PsA, while higher joint effusion scores were associated with lower odds of PsA. Enthesitis, tenosynovitis and BME/osteitis scores were not independently associated after adjustment. Prominent reticular vascular-like signal showed a strong exploratory association with PsA (Firth OR 85.02, 95% CI 15.07–479.83; P <0.001), but the wide CI reflects sparse cells and limited precision. Wrist synovitis (OR 0.08, 95% CI 0.02–0.26) and wrist bone erosion (OR 0.11, 95% CI 0.04–0.36) were associated with lower odds of PsA, favoring RA (both P <0.001) (**Table 1**).

### Individual-feature discrimination and reader reliability

The AUC for prominent reticular vascular-like signal was 0.902 (95% bootstrap CI 0.841–0.963). AUCs for RA-favoring wrist synovitis and wrist bone erosion were 0.808 (0.717–0.893) and 0.763 (0.664–0.852), respectively. AUCs were 0.782 (0.678–0.876) for periarthritis, 0.759 (0.651–0.862) for extracapsular soft tissue inflammation and 0.688 (0.568–0.799) for RA-favoring joint effusion (**Figure 2**). These are apparent individual-feature estimates from the same exploratory cohort, not externally validated diagnostic performance.

**Figure 2:**
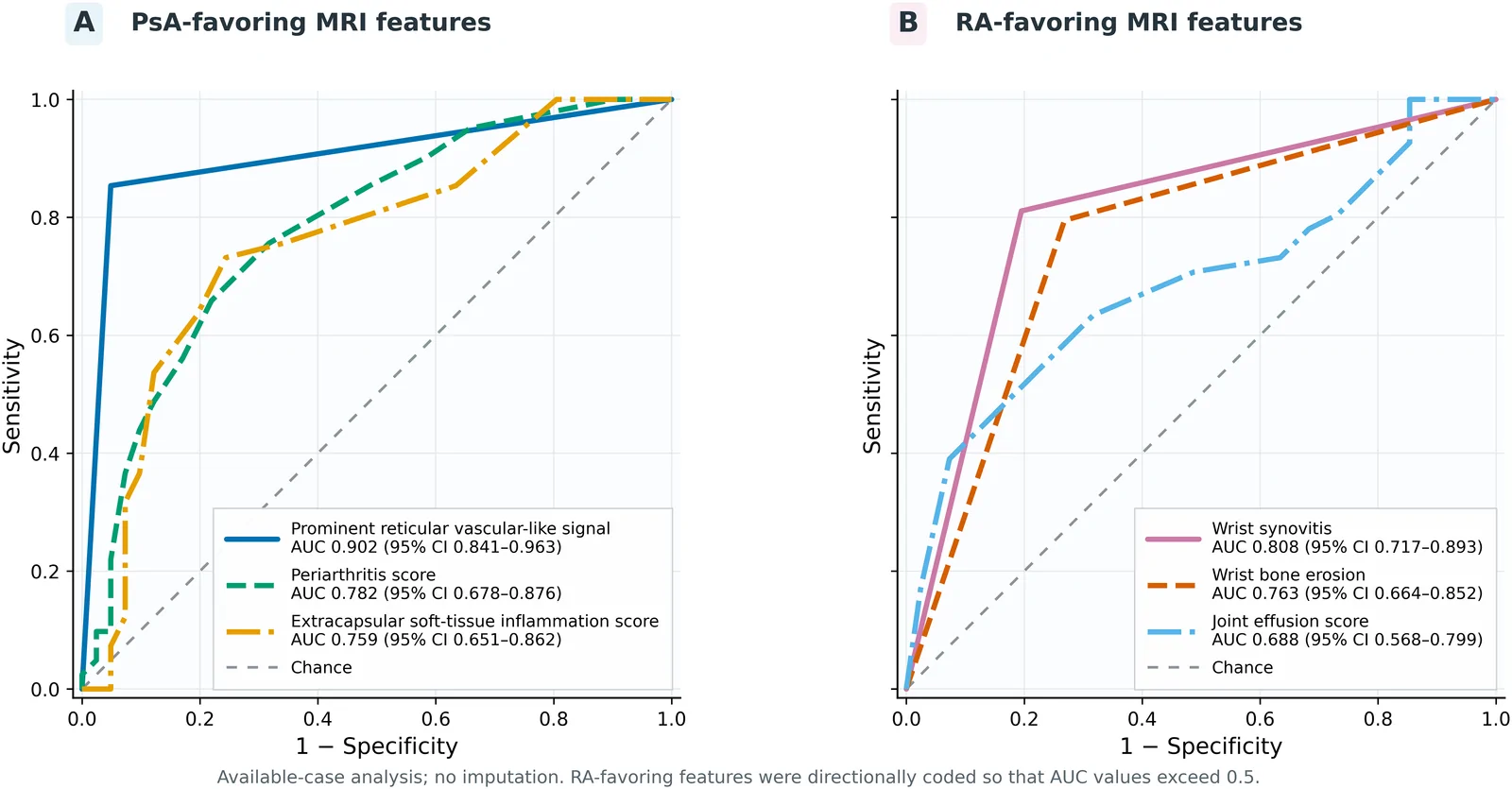
Receiver operating characteristic curves for selected individual MRI features. **(A)** PsA-favoring features: prominent reticular vascular-like signal, periarthritis score and extracapsular soft tissue inflammation score. **(B)** RA-favoring features: wrist synovitis, wrist bone erosion and joint effusion score. Apparent AUCs (95% group-stratified bootstrap CIs) were 0.902 (0.841–0.963) for prominent reticular vascular-like signal, 0.808 (0.717–0.893) for RA-favoring wrist synovitis, 0.763 (0.664–0.852) for RA-favoring wrist bone erosion, 0.782 (0.678–0.876) for periarthritis, 0.759 (0.651–0.862) for extracapsular soft tissue inflammation and 0.688 (0.568–0.799) for RA-favoring joint effusion. RA-favoring features were directionally coded so that AUC values exceeded 0.5. No combined diagnostic model is shown.

Across the 60-examination reliability subset, inter-reader ICC or kappa coefficients ranged from 0.574 to 0.914 and intra-reader coefficients from 0.846 to 0.968 (**Table 3**). Inter-reader agreement was moderate for bead-like peritendinous effusion (kappa 0.574) and prominent reticular vascular-like signal (kappa 0.659), whereas the standard scores and wrist variables showed higher agreement. These values support feasibility but also indicate that the study-defined features need further standardization.

**Table 3:** Inter- and intra-reader reliability in the 60-examination reassessment subset.

Feature	Statistic	N	Inter-reader	Intra-reader
BME/osteitis score	ICC (2,1)	60	0.877	0.956
Synovitis score	ICC (2,1)	60	0.914	0.968
Joint effusion score	ICC (2,1)	60	0.844	0.921
Tenosynovitis presence	Cohen’s kappa	60	0.785	0.895
Enthesitis presence	Cohen’s kappa	60	0.815	0.963
Prominent reticular vascular-like signal	Cohen’s kappa	60	0.659	0.846
Bead-like peri-tendinous effusion	Cohen’s kappa	60	0.574	0.927
Wrist synovitis	Cohen’s kappa	58	0.793	0.897
Wrist bone erosion	Cohen’s kappa	60	0.832	0.933

BME, bone marrow edema; ICC, intraclass correlation coefficient. ICC(2,1) denotes a two-way random-effects, absolute-agreement, single-measure coefficient. Binary features were assessed with unweighted Cohen’s kappa. Wrist synovitis had 58 evaluable paired examinations; other listed features had 60.

## Discussion

In this retrospective case-control study, PsA was associated with more extracapsular and enthesis-associated hand or wrist MRI findings, including soft tissue inflammation, periarthritis, bead-like peritendinous effusion and interphalangeal-region enthesitis. RA showed more joint effusion, wrist synovitis and wrist bone erosion. The study-defined prominent reticular vascular-like signal markedly separated the groups but had a wide adjusted CI and only moderate inter-reader agreement. These findings are exploratory imaging associations rather than a validated diagnostic rule.

The broad anatomical contrast is consistent with prior comparative imaging literature describing more periarticular or extracapsular inflammation in PsA and more joint-centered and wrist involvement in RA (1, 13–15, 23). Those differences are therefore not novel by themselves. The present study’s narrower contribution is the operationalization of two study-defined visual patterns, the separation of wrist synovitis from wrist bone erosion, the site-level description of enthesitis and the reporting of repeatability. The synovio-entheseal complex literature supports the anatomical plausibility of enthesis-, tendon- and capsular-associated inflammation but should not be interpreted as direct comparative evidence (16, 17).

Prominent reticular vascular-like signal showed the largest adjusted association and the highest individual-feature AUC. Histopathological and early-arthritis studies provide biological context for differences in synovial angiogenesis and vascular morphology (18–20), but they do not establish this non-contrast MRI appearance as a biomarker. The sparse 2/41 vs, 35/41 distribution produced a large OR and wide CI despite bias-reduced estimation. Moreover, inter-reader kappa was 0.659. The feature should therefore be described as a research observation requiring standardized training examples, testing across scanners and sequences, and prospective external validation; it should not be used by clinicians as a stand-alone diagnostic sign.

Bead-like peritendinous effusion was associated with PsA after adjustment. High-resolution MRI and anatomical work have implicated the flexor tendon pulley and synovio-entheseal complex in PsA and dactylitis (16, 17, 23, 24), which provides anatomical plausibility for a discontinuous peritendinous morphology. However, none of those studies validates the present bead-like definition. Its moderate inter-reader kappa also argues for caution. Patient-level dactylitis and the combined tenosynovitis-plus-dactylitis outcome were not analyzed because those outcomes could not be audited consistently from the retrospective patient-level record.

The higher joint effusion score in RA should be interpreted within the anatomy sampled in this study. RA also had substantially more wrist synovitis and wrist bone erosion, and the summed effusion score may consequently have been influenced by wrist-region weighting. This finding should not be generalized to large joints or to overall effusion propensity in PsA and RA. Differences in unmeasured composite disease activity and the marked imbalance in treatment exposure may also have contributed.

Several limitations are important. First, the original screening log was unavailable, so the initial denominator, exclusion counts and consecutive nature of recruitment could not be verified; selection bias is possible. Second, study groups were defined by contemporaneous clinical diagnoses. Although every PsA case met CASPAR criteria and 40 of 41 RA cases met the 2010 threshold under the conservative verification rules, one clinically diagnosed RA case had a reconstructed score of 5, leaving potential misclassification; excluding this case in a sensitivity analysis did not materially alter the findings. The contribution of routine clinical MRI to each original diagnosis could not be reconstructed, although the blinded research readings did not define study groups. Third, DAPSA and DAS28 could not be reconstructed, and ESR/CRP do not replace composite activity measures. Treatment differed between groups, while dose, duration and indication were unavailable. Fourth, variable field of view limited distal interphalangeal evaluation and prevented a dedicated ulnocarpal score. Fifth, multiple exploratory comparisons were performed in a small single-center cohort without multiplicity correction or external validation. Finally, although repeatability was evaluated in 60 examinations, moderate agreement for the two study-defined features and the lack of prospective standardized acquisition limit immediate clinical translation.

In conclusion, this exploratory cohort showed an extracapsular and enthesis-associated hand or wrist MRI pattern favoring PsA and a joint-dominant pattern, including joint effusion and wrist synovitis or erosion, favoring RA. Prominent reticular vascular-like signal and bead-like peritendinous effusion are study-defined observations that require independent replication and improved standardization before they can inform routine diagnostic practice.

## Data Availability

The original contributions presented in the study are included in the article/supplementary material. Further inquiries can be directed to the corresponding authors.
